# Blocking key mutated hotspot residues in the RBD of the omicron variant (B.1.1.529) with medicinal compounds to disrupt the RBD-hACE2 complex using molecular screening and simulation approaches†

**DOI:** 10.1039/d2ra00277a

**Published:** 2022-03-04

**Authors:** Abbas Khan, AsfandYar Waheed Randhawa, Ali Raza Balouch, Naila Mukhtar, Abrar Mohammad Sayaf, Muhammad Suleman, Taimoor Khan, Shahid Ali, Syed Shujait Ali, Yanjing Wang, Anwar Mohammad, Dong-Qing Wei

**Affiliations:** Department of Bioinformatics and Biological Statistics, School of Life Sciences and Biotechnology, Shanghai Jiao Tong University Shanghai 200240 P.R. China dqwei@sjtu.edu.cn; Central Park Medical College Lahore Pakistan; Department of Botany, University of Okara Punjab Pakistan; School of Chemical Sciences, Universiti Sains Malaysia Pulau Pinang 11800 Malaysia; Centre for Biotechnology and Microbiology, University of Swat Kanju Khyber Pakhtunkhwa Pakistan; Engineering Research Center of Cell and Therapeutics Antibody, School of Pharmacy, Shanghai Jiao Tong University Shanghai 200240 P.R. China; Department of Biochemistry and Molecular Biology, Dasman Diabetes Institute Dasman Kuwait; State Key Laboratory of Microbial Metabolism, Shanghai-Islamabad-Belgrade Joint Innovation Center on Antibacterial Resistances, Joint Laboratory of International Laboratory of Metabolic and Developmental Sciences, Ministry of Education and School of Life Sciences and Biotechnology, Shanghai Jiao Tong University Shanghai 200030 P.R. China; Peng Cheng Laboratory Vanke Cloud City Phase I Building 8, Xili Street, Nashan District Shenzhen Guangdong 518055 P.R China

## Abstract

A new variant of SARS-CoV-2 known as the omicron variant (B.1.1.529) reported in South Africa with 30 mutations in the whole spike protein, among which 15 mutations are in the receptor-binding domain, is continuously spreading exponentially around the world. The omicron variant is reported to be highly contagious with antibody-escaping activity. The emergence of antibody-escaping variants is alarming, and thus the quick discovery of small molecule inhibitors is needed. Hence, the current study uses computational drug screening and molecular dynamics simulation approaches (replicated) to identify novel drugs that can inhibit the binding of the receptor-binding domain (RBD) with hACE2. Screening of the North African, East African and North-East African medicinal compound databases by employing a multi-step screening approach revealed four compounds, namely (−)-pipoxide (C1), 2-(*p*-hydroxybenzyl) benzofuran-6-ol (C2), 1-(4-hydroxy-3-methoxyphenyl)-2-{4-[(*E*)-3-hydroxy-1-propenyl]-2-methoxyphenoxy}-1,3-propanediol (C3), and Rhein (C4), with excellent anti-viral properties against the RBD of the omicron variant. Investigation of the dynamics demonstrates stable behavior, good residue flexibility profiles, and structural compactness. Validation of the top hits using computational bioactivity analysis, binding free energy calculations and dissociation constant (*K*_D_) analysis also indicated the anti-viral properties of these compounds. In conclusion, this study will help in the design and discovery of novel drug therapeutics, which may be used against the emerging omicron variant of SARS-CoV-2.

## Abstract 2

A new variant of SARS-CoV-2 known as the omicron variant (B.1.1.529) reported in South Africa with 30 mutations in the whole spike protein, among which 15 mutations are in the receptor-binding domain, is continuously spreading exponentially around the world.

## Graphical abstract

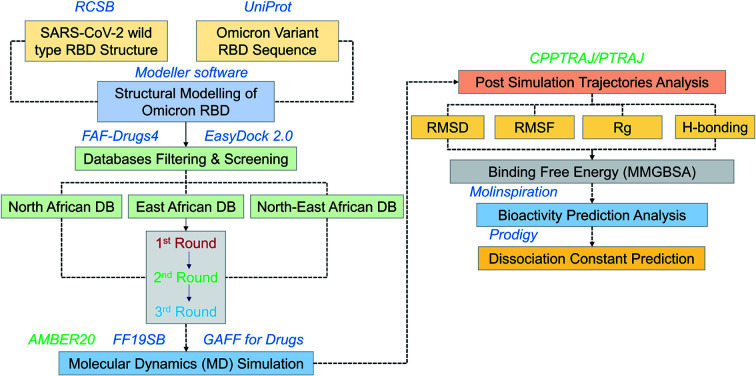

## Introduction

The pandemic instigated by a member of the coronavirus family, known as SARS-CoV-2, in late 2019 continues to spread chaos around the world. Since the emergence of the first case of COVID-19, many variants of the virus have been reported, increasing the risk of reinfection and immunity evasion.^1^ To date, many variants classified as either variants of concern (VOCs) or variants of interest (VOIs) have been reported to differentially challenge public health. Among the reported variants, the alpha, beta, gamma, delta, and omicron variants have been classified as VOCs.^2^ The alpha variant, also known as B.1.1.7, reported in almost 120 countries and harboring 69–70del, N501Y and P681H mutations in the spike proteins, was reported to increase transmissibility by 40–80%.^3^ Later on, a new version of B.1.1.7 was reported, known as B.1.1.7+ E484K, with an extra mutation in the receptor-binding domain (RBD) of the spike protein along with N501Y. This variant was reported only in 39 confirmed cases and then disappeared.^4^ The beta variant (B.1.351), reported in South Africa with mutations K417N, E484K and N501Y, was confirmed to increases the transmissibility as well as cause reduction in T cells and other immune responses triggered against COVID-19 infection. In January 2021, a new variant, P.1, was reported in Brazil harbouring K417T, E484K and N501Y mutations in the RBD of the spike protein. This variant increased the transmission by 38%, mortality by 50% and the immune response against reinfection was also reduced.^5^ Abbas *et al.* used structural modeling and protein coupling approaches to discover the mechanism of higher infectivity caused by these variants of SARS-CoV-2.^6–8^ They reported that the binding affinity for the host receptor hACE2 increased while the electrostatic interactions were considered the prime factors for enhanced binding. A more devastating variant of SARS-CoV-2 was reported in India in Oct 2020 and officially named B.1.617.2. This variant harbours L452R, T478K and P681R mutations in the RBD with an 87% increase in transmissibility, 85% increment in hospitalization and a mortality increase of 137%. Later on, a new version of this variant known as “delta plus” having an extra mutation, K417N, was reported in India and UK.^9^ Likewise, a new variant known as Mu (μ) or B.1.621 was reported to contain mutations in the spike protein among which some are shared with other VOC. The novel mutations in this variant include R346K, Y144T, Y145S, and insertion at 146N position.^10^ A novel VOI termed as C.37 or λ variant exhibit L452Q and F490S mutations in RBD, which consequently reduces the antibody mediated neutralization.^11^ On the other hand, the B.1.617.1 variant reported having L452R mutation in the RBD is linked with altered antibody neutralization by disrupting the respective conformational epitopes. Moreover, the VOI Iota (ι) (lineage B.1.526) also contains mutation E484K showing resistance to therapeutic monoclonal antibodies and is less susceptible to neutralization.^7^

The latest variant, designated as the omicron variant and officially named as B.1.1.529, reported in November 2021 in South Africa, harbours 30 mutations in the spike protein (A67V, Δ69–70, T95I, G142D, Δ143–145, Δ211, L212I, ins214EPE, G339D, S371L, S373P, S375F, K417N, N440K, G446S, S477N, T478K, E484A, Q493R, G496S, Q498R, N501Y, Y505H, T547K, D614G, H655Y, N679K, P681H, N764K, D796Y, N856K, Q954H, N969K, L981F), among which, 15 mutations lie in the RBD.^12^ Among the others, such as nsp3 (K38R, V1069I, Δ1265, L1266I, A1892T), nsp4 (T492I), nsp5 (P132H), nsp6 (Δ105–107, A189V), nsp12 (P323L), while I42V was reported in NSP14. Three sub-lineages of this variant such as BA.1/B.1.1.529.1, BA.2/B.1.1.529.2 and BA.3/B.1.1.529.3 are circulating. WHO (World Health Organization) reported this variant as the most serious threat to public health that evades the immune response.^13^ The variant poses a significant health threat to the world and the therapeutic efficacy of already developed vaccine therapeutics against it remains elusive.^14,15^ Further investigations are required to depict the molecular mechanism of pathogenicity related to the omicron variant. Moreover, improved strategies are needed to design novel therapeutics against the newly emerged SARS-CoV-2 variants.

To deal with this worrisome pandemic situation, further research is needed for the rapid development of safe and effective therapeutics. In this regard, the spike protein is deemed as the most effective druggable target for the development of drugs for COVID-19 treatment.^16,17^ The increasing prevalence of mutations in the RBD of SARS-CoV-2, which is linked with an enhanced viral affinity for host receptors and pathogenicity, is crucial to be considered as a therapeutic target. Thus, herein, we employed computational molecular screening against the RBD of the omicron (B.1.1.529) variant and evaluated the binding affinity of drug-like molecules against it. The current study uses *in silico* approaches such as molecular docking, simulation and free energy calculations for the identification of phytomedicines from North African, East African and North-East African medicinal compounds databases against the SARS-CoV-2. The results demonstrate critical information about the anti-viral potency of the screened drug-like molecules against the mutations in the RBD domain of the omicron variant. The study will help in the design and discovery of novel drug therapeutics, which may be deployed against the emerging omicron variant of SARS-CoV-2.

## Materials and methods

### Structures retrieval and modelling

The wild-type structure of the RBD was obtained from RCSB using 6M0J PDB ID.^18^ The sequence of RBD was retrieved from UniProt, which ranges from 313–526.^19^ Fifteen mutations reported in the RBD of omicron variants were manually integrated into the primary sequence and subjected to molecular modelling. A PSI-BLAST was carried out, which also results in 6M0J as the closest template, which shares a higher identity with the omicron RBD. A Modeller program was used for homology modeling of the RBD of the omicron variant of SARS-CoV-2.^20,21^ The modelled structure was minimized, relaxed, prepared and cleaned for virtual screening.

### Virtual drugs screening and re-scoring of the top hits

North African, East African and North-East African were downloaded from the African Natural Products Databases (ANPDB) website (http://african-compounds.org/anpdb/) in a 3D-SDF format which were prepared for screening.^22^ These databases are a collection of natural products from South African natural compounds, which have diverse medicinal features. Prior to the computational screening of these databases, the FAF-Drugs4 webserver was used to obtain only for non-toxic, drug-like molecules obeying Lipinski's rule of five.^23^ The filtered databases were then screened against the interface site of the RBD. All the drugs were converted to .pdbqt format prior to screening. For virtual drug screening EasyDock Vina 2.0, a GUI interface for virtual screening of the three databases was used. EasyDock Vina uses the AUTODOCK4 algorithm to screen and order the best drug-like molecules. In the initial stage, 16 exhaustiveness was used for quick screening. The top-scoring compounds were then selected again and screened using 64 exhaustiveness. The purpose of the second screening was to remove false-positive results and re-evaluate the best compounds. The top 10% from each database were then selected for induced-fit docking (IFD) using AutoDockFR, which enables receptor flexibility and also supports covalent docking.^24^ This approach also uses AutoDock4 and Vina modes for docking but is very fast and accurate. The final best hits were then subjected to bioactivity evaluation, dissociation constant and molecular dynamics simulation analysis.

### Dynamic evaluation of the final drugs-RBD complexes

The obtained top natural compounds in complex with the omicron RBD were tested for dynamic feature evaluation using AMBER20 package solvated with OPC water model. For this purpose, the FF19SB force field and (GAFF) “General Amber Force Field” were used to parameterize both the protein and small drug molecules, respectively.^25,26^ Prior to force field parametrization, the drug topologies were generated with an antechamber module. To neutralize the effect of any charge Na^+^ and Cl^+^ ions were inoculated followed by energy minimization in two steps (algorithms: steepest descent and conjugate gradient) was achieved. Next, the heating and equilibration steps were completed. Finally, for each complex 200 ns (two replicas) of the production run was completed. To treat the long-range electrostatic interactions with a 10.0 Å cutoff distance, the particle mesh Ewald algorithm was used. However, the covalent bonds, if any, were treated with the SHAKE algorithm. Finally, the CPPTRAJ package was used to analyze the trajectories while the PMEMD.cuda was used for running the simulations.^27^

### Post-simulation validation of the top hits

#### The binding free energy calculations

The evaluation of the strength of small molecules binding through binding free energy (BFE) using MM/GBSA is the most widely employed approach used in different research investigations. Keeping in view the importance of this approach in re-ranking the binding conformations, we also used the MMPBSA.py script to compute the binding free energy of the RBD-ligand complexes by considering 2500 snapshots. For this purpose, the following equation was used to estimate the BFE:Δ*G*_bind_ = Δ*G*_complex_ − [Δ*G*_receptor_ + Δ*G*_ligand_]

The Δ*G*_bind_ represent the total binding energy, while Δ*G*_receptor_, Δ*G*_ligand_, and Δ*G*_complex_ represent the binding energy of protein, drug, and complex, respectively. The following equation was used to estimate the individual binding energies like bonded (*G*_bond_), electrostatic (*G*_ele_), polar (*G*_pol_) and non-polar (*G*_npol_), which contribute to the total binding free energy.*G* = *G*_bond_ + *G*_electrostatic_ + *G*_van der Waal_ + *G*_polar_ + *G*_non-polar_

### Prediction of bioactivity and determination of *K*_D_ (dissociation constant)

To estimate the IC_50_ value as a bioactivity predictor each shortlisted compound was subjected to a cheminformatics tool, Molinspiration (https://www.molinspiration.com/cgi-bin/properties). Hundreds of studies have leveraged molinspiration to forecast bioactivity values.^28–32^ In addition, the PRODIGY (PROtein binDIng enerGY) (https://wenmr.science.uu.nl/prodigy/) webserver was utilized to calculate the dissociation constant values for various biological complexes in order to offer convincing information of the strength of binding as *K*_D_.^33,34^

## Results and discussion

**Fig. 1:**
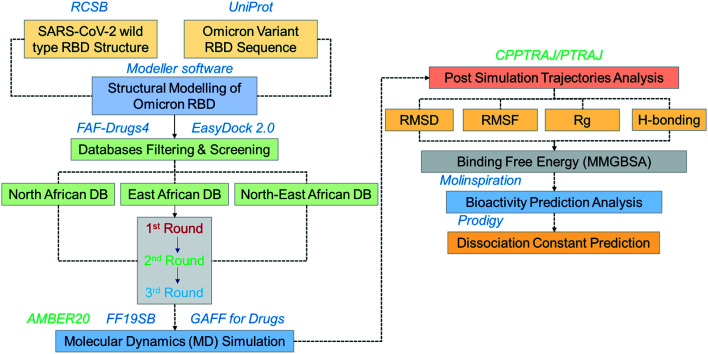
Overall workflow of this work. The figure explains the structural modeling, screening, simulation, post-simulation and validation steps.

The emergence of the omicron variant has created alarming situations around the world and worsened the pandemic situation further. It has been reported that this variant is more transmissible and increases the chances of re-infection. Despite all these, the evasion of almost all the neutralization antibodies and reduction in the efficacy of the different vaccines is a greater threat.^35^ Thus, further insightful studies are required to overcome the problem of vaccine therapeutic evasion. Thus, small molecules development is of great interest for effective treatment. Hence in this study, we used computational drugs screening and simulation approaches to identify potential drugs for omicron (B.1.1.529) RBD. Screening of multiple databases, *i.e.* North African, East African and North-East African databases shortlisted potential anti-viral drugs. The overall workflow of the work is given in Fig. 1.

**Fig. 2:**
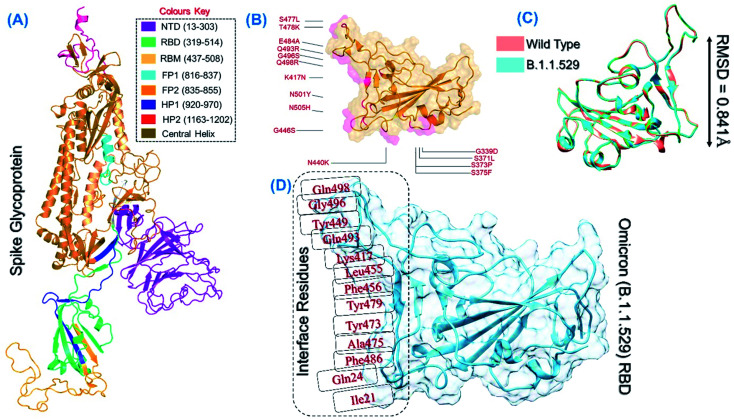
(A) Structural organization of the whole spike protein. (B) RBD domain of the B.1.1.529 variant with the reported mutations. (C) Superimposed wild type and B.1.1.529-RBD structures. (D) Interface residues on RBD used for grid generation and screening.

The spike protein is an essential determinant of the COVID-19 infection initiation and entry into the host cell. It is a multi-domain protein with the most important RBD, which comes in direct contact with the host receptor and fuses with the cellular membrane for entry. It is deemed an important drug and vaccine target for the neutralization of the virus. The structure of the spike protein and its domain organization is shown in Fig. 2A. The structure of RBD of the omicron variant was modelled and the active site residues were specified for docking. The mutations in the RBD of the omicron variant are shown in Fig. 2B. The superimposed structure of the wild type and omicron RBD revealed 0.841 Å of RMSD difference, which demonstrates that the new variant acquired conformational variations through mutations fitness (Fig. 2C). Prior to the screening of the databases, the interface residues shown in Fig. 2D were considered to generate the receptor grid (6.04 × −70.97 × 24.028).

Prior to database screening, filtering of drugs-like molecules using Lipinski's rule of five was performed. Among the total 1871 compounds in the East African database, only 1131 compounds were selected to follow the R5. In the North African database, a total of 4924 compounds were screened for R5 rules and only 357 compounds were excluded. In the northeast African database among 6512, only 5422 compounds were accepted to obey Lipinski's rule of five. Thus, we screened a total of 11 102 compounds by using a multi-step screening approach. The first step of virtual screening results in docking scores ranging from −4.5 to −2.5 kcal mol^−1^. Among these, 2664 compounds reported a docking score less than −3.0 while 8438 reported more than −3.0 kcal mol^−1^. Among 2664, only the top 20% of compounds (266) were selected for the third round of screening. An induced-fit docking approach was then employed to screen the top 418 compounds, which resulted in docking scores ranging from −9.63 to −6.71 kcal mol^−1^. Among these, only the top ten were selected for the docking using Auto Dock Vina. Among the 42 compounds (10%), only seven compounds were reported to have a docking score higher than −5.0 kcal mol^−1^ and good interaction profiles. Among the seven compounds (−)-pipoxide (C1), 2-(*p*-hydroxybenzyl) benzofuran-6-ol (C2), 1-(4-hydroxy-3-methoxyphenyl)-2-{4-[(*E*)-3-hydroxy-1-propenyl]-2-methoxyphenoxy}-1,3-propanediol (C3), and Rhein (C4) were selected for further analysis. The top four compounds were selected for detailed investigation and are given in Table 1.

**Table 1:** List of the top four hits with their scientific names, 2D structures and docking scores

S. no.	Compound name	2D structure	Docking score
1	(−)-Pipoxide	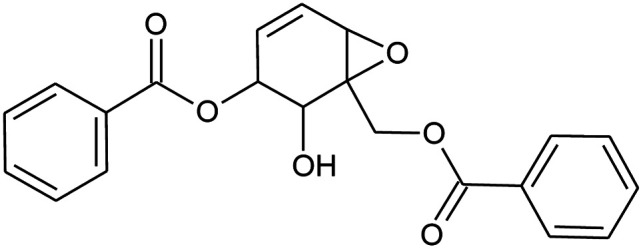	−5.794
2	2-(*p*-Hydroxybenzyl)benzofuran-6-ol	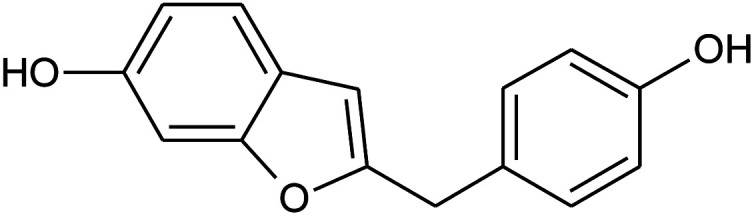	−5.124
3	Rhein	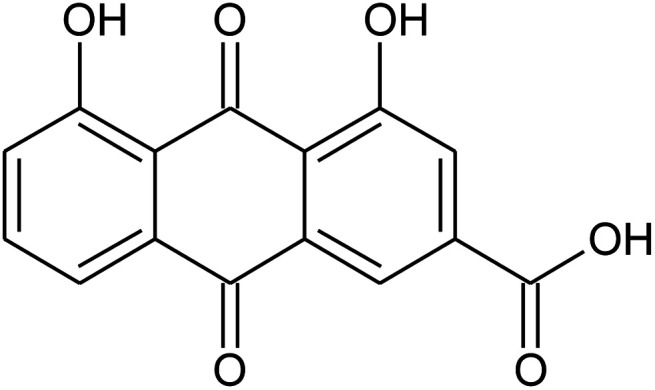	−5.127
4	1-(4-Hydroxy-3-methoxyphenyl)-2-{4-[(*E*)-3-hydroxy-1-propenyl]-2-methoxyphenoxy}-1,3-propanediol	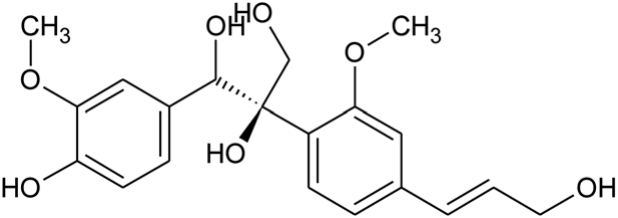	−7.601

**Fig. 3:**
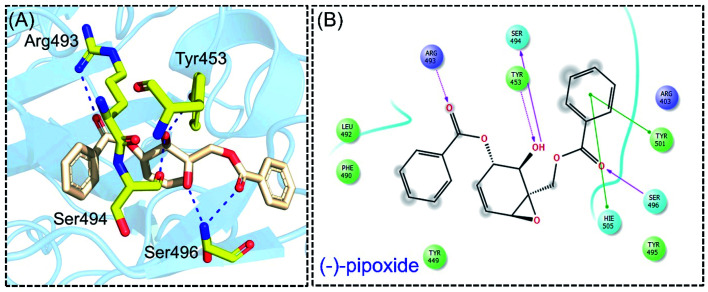
Interaction pattern of (−)-pipoxide with the RBD of the omicron variant. (A) 3D interaction pattern of (−)-pipoxide; (B) the 2D interaction pattern generated with Maestro free Academic version 2018-1 (for visualization only).

**Fig. 4:**
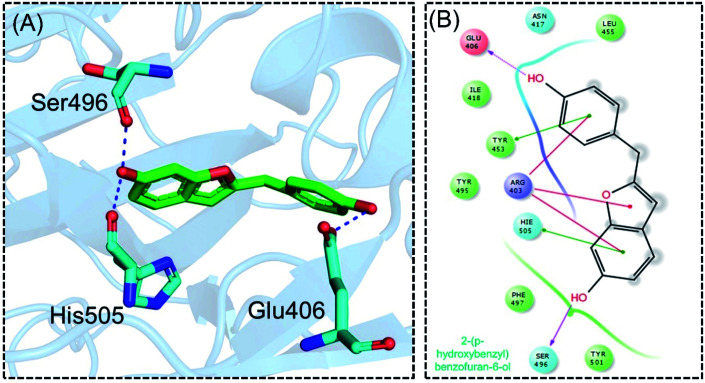
Interaction pattern of 2-(*p*-hydroxybenzyl) benzofuran-6-ol with the RBD of the omicron variant. (A) 3D interaction pattern of 2-(*p*-hydroxybenzyl) benzofuran-6-ol; (B) the 2D interaction pattern generated with Maestro free Academic version 2018-1 (for visualization only).

Detailed interaction analysis of the top four compounds revealed information about hydrogen bonding, pie–pie interactions, salt-bridges and pie-stacking. In the case of C1, a total of five hydrogen bonds with Tyr453, Arg493, Ser494 and Ser496 were established. Among the others, Tyr501 and His505 also formed pie–pie interactions. It can be seen that this compound blocked the key mutated residues such as Arg493, Ser494, Ser496, Tyr501 and His505, which consequently halted the binding of RBD to the hACE2. The docking score for (−)-pipoxide was reported to be −5.78 kcal mol^−1^. The 3D interaction pattern of C1 is given in Fig. 3A while the 2D interactions are shown in Fig. 3B. C2 on the other hand with the docking score −5.12 kcal mol^−1^ established three hydrogen bonds, including Glu406, Ser496 and His505. Tyr453 and His505 also reported two pie–pie stacking contact with the ligand. Moreover, the three pie–cation interactions are formed by the only residue Arg493, which is mutated in the omicron variant. Herein, the same residues are targeted, which are novel mutations in the RBD of the omicron variant. The 3D interaction pattern of C2 is given in Fig. 4A while the 2D interactions are shown in Fig. 4B.

**Fig. 5:**
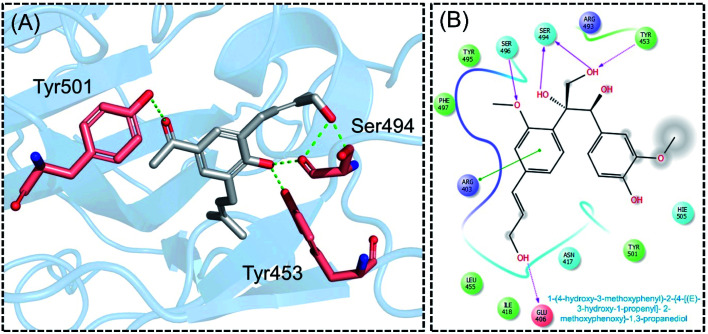
Interaction pattern of C3 with the RBD of the omicron variant. (A) 3D interaction pattern of C3; (B) the 2D interaction pattern generated with Maestro free Academic version 2018-1 (for visualization only).

Next, the binding pattern of C3 demonstrated the strongest binding of the other three compounds. The docking scores of −7.601 kcal mol^−1^ for five hydrogen binds and one pie–pie interaction were reported. The residues Glu406, Tyr453, Ser494 and Ser496 established hydrogen bonds while the only pie–pie interaction was established with the C3. The interaction pattern of C3 is similar to those of C1, C2 and C4, which also block these residues to disrupt the binding between RBD and hACE2. The 3D interaction pattern of C3 is given in Fig. 5A while the 2D interactions are shown in Fig. 5B.

**Fig. 6:**
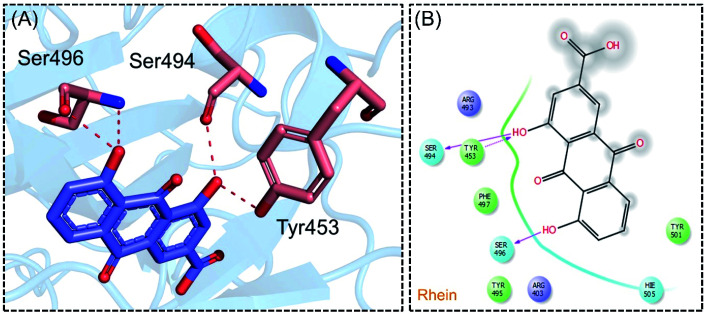
Interaction pattern of Rhein with the RBD of the omicron variant. (A) 3D interaction pattern of Rhein; (B) the 2D interaction pattern generated with Maestro free Academic version 2018-1 (for visualization only).

The molecular interaction analysis of C4 reported only four hydrogen bonds with Tyr453, Ser494 and Ser496. The docking score for C4 was reported to be −5.127 kcal mol^−1^. The interaction pattern of C4 is similar to those of C1 and C2, which also block these residues to disrupt the binding between RBD and hACE2. The 3D interaction pattern of C4 is given in Fig. 6A while the 2D interactions are shown in Fig. 6B.

### Dynamic and binding stability analysis

**Fig. 7:**
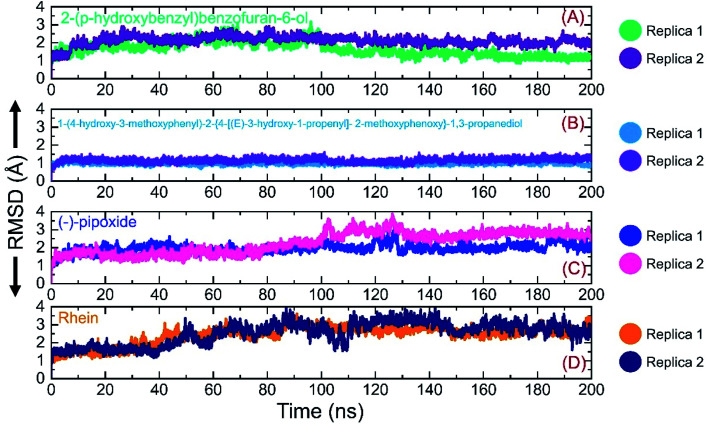
Binding stability analysis of the top complexes C1–C4. (A) RMSD of C1 during simulation. (B) RMSD of C2 during simulation. (C) RMSD of C3 during simulation. (D) RMSD of C4 during simulation.

Investigation of dynamic stability using simulation trajectories is important to reveal the binding of the small molecular ligand in the binding cavity. It is essential to demonstrate the dynamic stability of the interacting molecules to determine the binding strength. Hence, here, we also calculated the dynamic stability of the top complexes as root mean square deviation (RMSD). The stability of each complex was calculated as a function of time using the simulation trajectory. As given in Fig. 7A, the C1-RBD demonstrated stable dynamics and no significant deviation was observed. The complex reached stability at 1.68 Å and equilibrium at 3 ns. The RMSD of this complex increased a little between 22–30 ns and then again decreased back with a uniform pattern until 80 ns. A smaller increase in the RMSD was experienced between 81–88 ns and then again the RMSD decreased. During the last 100 ns (101–200 ns) the RMSD decreased gradually and was stabilized in the long-run simulation. No significant convergence was observed during the last 100 ns. Similarly in replica 2 of the C1 complex, a more similar trend in the RMSD graph with few deviations until 80 ns was observed. However, the RMSD then decreased gradually and followed a similar pattern as that of replica 1. The complex demonstrated a stable binding during the simulation and the average RMSD for this complex replica 1 was reported to be 1.63 Å while for replica 2 the average RMSD was calculated to be 1.65 Å. The RMSD graph of C1 is given in Fig. 7A. On the other hand, the RMSD graph of C2 demonstrated a more stable behavior comparatively but overall the complex remained stable. The structure gained stability at 1.2 Å and then a uniform pattern of RMSD was followed. The complex reported minor deviations such as between 61–72 ns and 91–95 ns but no major convergence was observed. The average RMSD for C2 was reported to be 1.22 Å for replica 1, while for replica 2, a similar average RMSD was reported. The RMSD graph of C2 is given in Fig. 7B. Furthermore, the RMSD of C3 also reported a little unstable dynamic behavior and reported only minor acceptable deviations. In both replicas, 1 and 2, the complex initially demonstrated a stable uniform pattern until 100 ns, however, at 101–130 ns both the replicas reported an increase in the RMSD and minor structural perturbation was reported. Afterwards, both the replicas gained stability again and no significant deviation was observed particularly in replica 1. Though replica 2 demonstrated a little deviation in the later part of simulation overall the average RMSD remained lower than replica 1. The complex reached stability at 1.9 Å and the pattern continues until 100 ns of simulation. The average RMSD for C3 was reported to be 1.96 Å for replica 1, while for replica 2, it was reported to be 1.95 Å. The RMSD graph of C3 is given in Fig. 7C. Finally, the RMSD of C4 was also demonstrated to see the binding strength of the Rhein compound. As shown in Fig. 7D, the complex remained stable, however, an increment in the RMSD value over the simulation time was observed. The structure did not report any significant perturbation except a decrease–increase pattern between 40–100 ns. The RMSD in both replicas increased gradually after 40 ns and continued to follow this pattern until 90 ns. The average RMSD for C4 was reported to be 2.84 Å for replica 1 while for replica 2 the average RMSD was 2.90 Å. The RMSD graph of C4 is given in Fig. 7D. Overall the simulation of these complexes reported stronger binding and consequently potential robust inhibitory properties against the RBD of omicron and other variants. These results explain the strong inhibitory features, of C1 and C2 particularly, owned by the shortlisted drugs.

### Structural compactness analysis

**Fig. 8:**
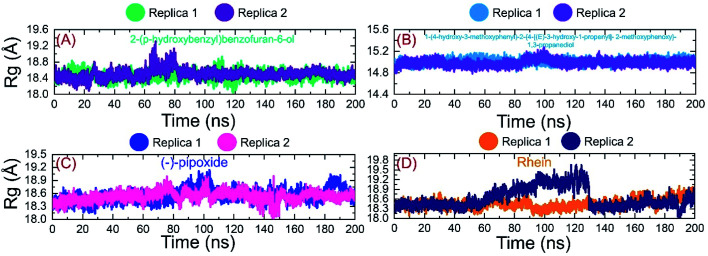
Structural compactness analysis of the top complexes C1–C4 calculated as *R*_g_. (A) *R*_g_ of C1 during simulation. (B) *R*_g_ of C2 during simulation. (C) *R*_g_ of C3 during simulation. (D) *R*_g_ of C4 during simulation.

Next, we evaluated the structural compactness of each complex in a dynamic environment to reveal the binding and unbinding events that occurred during the simulation. Herein, the structural compactness was computed as the radius of gyration (*R*_g_) as a function of time. As given in Fig. 8A, the *R*_g_ of C1 followed a similar pattern as RMSD. The complex initially reported higher *R*_g_ values for a shorter period between 8–16 ns and then the *R*_g_ value decreased back and continues to follow a uniform pattern until 100 ns. In the extended simulation until 200 ns a uniform *R*_g_ pattern was observed in replica 1. Replica 2 reported an increment in the *R*_g_ value between 60–80 ns and then decreased back. A uniform pattern was followed until 200 ns with no significant increase or decrease in the *R*_g_ value. The average *R*_g_ value for C1 was calculated to be 18.4 Å for replica 1 while for replica 2 the average *R*_g_ value was 18.0 Å. Likewise, the C2 complex demonstrated a similar *R*_g_ pattern as RMSD, thus determining the tighter binding of C2 during the simulation. The average *R*_g_ values for C2 were reported to be 15.0 and 15.1 Å for replica 1 and replica 2, respectively. On the other hand, C3 demonstrated a little perturbation in the *R*_g_ value, particularly between 1–20 ns and then despite a gradual increment in a uniform pattern was reported. The *R*_g_ for C3 (replica 1 and replica 2) demonstrated a little perturbation between 80–100 ns and then no significant convergence was experienced. The C4 complex also reported a more similar pattern to RMSD. The complex initially reported a flattened graph until 40 ns, then a little decrease and increased at 65 ns. The *R*_g_ then decreased again in the end. The average *R*_g_ for C4 was reported to be 18.42 Å. Conclusively these results report a stable binding of the ligand on the binding surface with minimal unbinding events and favourable inhibition properties. The *R*_g_ graphs of C1–C4 (both replicas) are shown in Fig. 8A–D.

### Residual flexibility analysis

**Fig. 9:**
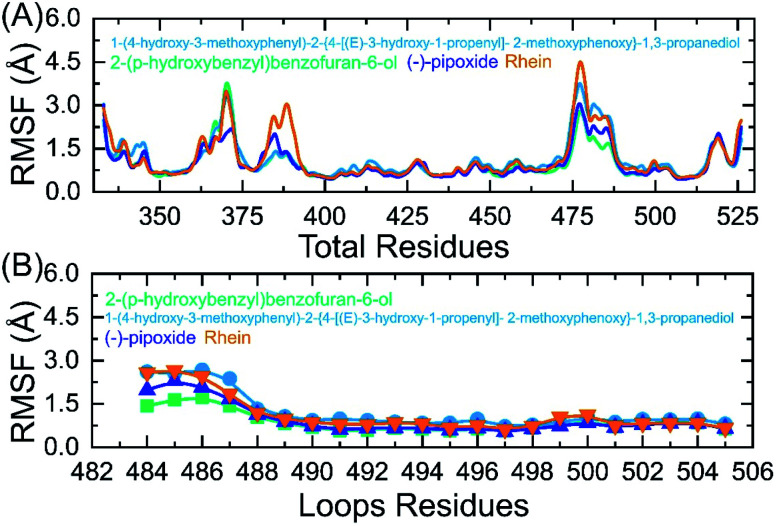
(A) Residual flexibility analysis of C1, C2, C3 and C4-RBD complexes. (B) Flexibility index of the three binding loops (484–505) required for interaction.

An insight into the residue-level fluctuations of the wild and the variants was further accomplished as such local level flexibility conferred strength to intermolecular binding, negatively impacting molecular recognition, and can potentially influence the overall function of the biological molecule. Herein, we calculated the residual flexibility as root mean square fluctuation (RMSF). Higher and lower RMSF implies flexible and stable regions, respectively (Fig. 9A). All the complexes here demonstrated a similar pattern of residual flexibility. All the complexes except C3 reported a higher RMSF for region 360–375 then the C4 complex only demonstrated a higher RMSF for region 378–385. The region between 472–482 in all the complexes also demonstrated a higher RMSF. Previously the three loops shown in Fig. 9B were reported to confer an important role in the higher affinity and increased transmissibility. Abbas *et al.* reported that the three loops reported higher flexibility in almost all the variants thus explaining the importance of these loops in the binding.^7,8,36^ It can be seen that the residual flexibility index of these loops is decreased and thus reduces the chances of binding with hACE2. This implies that the binding of these ligands produces different internal residual dynamics patterns and causes to stabilize the movement of loops required for direct interaction with hACE2 to minimize the chances of coupling with the receptor. For replica 2 all the complexes demonstrated a more similar pattern of flexibility but a little higher fluctuation in each complex was witnessed. The RMSF graphs of these complexes replica 2 are given in ESI Fig. S1A and B.†

### Hydrogen bonding analysis

**Fig. 10:**
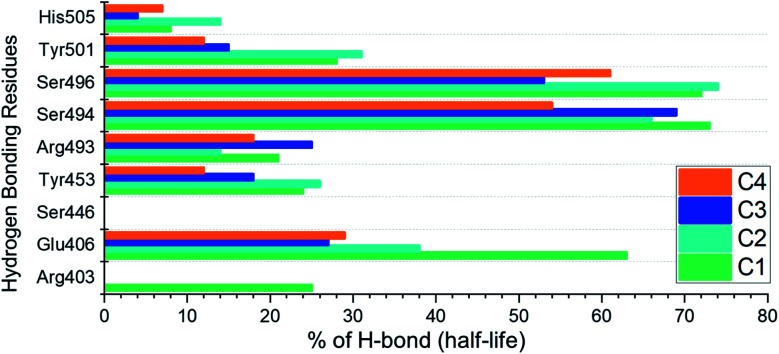
Hydrogen bonding half-life estimation for the C1–C4 complexes during the 200 ns simulation.

Analysis of hydrogen bonds demonstrates the binding strength of the interacting molecules. Hence, to observe how the hydrogen bonding pattern changed over the simulation, we calculated the total number of hydrogen bonds in each trajectory. The calculated hydrogen bonds for each complex as a function of time are given in ESI Fig. S2.† Moreover, we also calculated the hydrogen bonding half-life for each interacting residue in the complex. The interacting residues include Arg403, Glu406, Ser446, Tyr453, Arg493, Ser494, Ser496, Tyr501 and His505. The half-life analysis of the hydrogen bonds revealed that Glu406, Tyr453, Arg493, Ser494 and Ser496 contributed mainly towards the tighter binding while in the case of C1 and C2 the extra strength to the binding is conferred by Tyr501. The hydrogen bonding percentage of each residue in each complex is shown in Fig. 10.

### Binding free energy estimation

The binding strength of small molecules employing the binding free energy method, *i.e.* MM-GBSA is a widely applicable approach to re-demonstrate the docking stability and correct binding. The MM-GBSA approach, mentioned above, is more computationally affordable than the costly alchemical free energy method. It is one of the accurate methods by comparing with the rational scoring functions. Keeping in mind, the applications of this method, we also utilized the same approach to estimate the binding free energy for C1, C2, C3 and C4-RBD complexes. The total binding free energy (TBFE) for the C1-RBD complex was estimated to be −60.38 kcal mol^−1^, for C2-RBD the TBFE was −41.44 kcal mol^−1^, for C3-RBD complex −36.22 kcal mol^−1^ while C4-RBD complex the TBFE −44.30 kcal mol^−1^. The vdW values for C1-RBD, C2-RBD, and C3-RBD and C4-RBD complexes were reported to be −51.25, −36.72, −31.85 and −45.77 kcal mol^−1^, respectively. For these complexes, the electrostatic energy was reported to be −28.14, −20.51, −17.48 and −23.75 kcal mol^−1^, respectively. Decisively, this demonstrates that these small molecules effectively bind to the interface residues of RBD robustly and could potentially hinder the interaction with the hACE2. The binding free energy results are given in Table 2.

**Table 2:** MM/GBSA results for all the complexes including vdW, electrostatic and the total binding energy. All the energies are given in kcal mol^−1^

Complexes names	vdW	Electrostatic	SA	GB	Total
C1-RBD	−51.25	−28.14	−16.32	35.33	−60.38
C2-RBD	−36.72	−20.51	−6.55	22.34	−41.44
C3-RBD	−31.85	−17.48	−13.52	26.63	−36.22
C4-RBD	−45.77	−23.75	−11.36	36.58	−44.30

### Bioactivity prediction and dissociation constant (*K*_D_)

Estimation of IC_50_ values for different complexes of druggable proteins is commonly performed using *in silico* bioactivity prediction. Using the molinspiration server, we calculated the bioactivity scores as: C1-RBD (0.39), C2-RBD (0.21), C3-RBD (−0.31) and C4-RBD (0.26), respectively, which, demonstrate the stronger bioactivity against the RBD protein. For re-ranking of protein-ligand complexes using *K*_D_ and validation of potential inhibitory properties of these final compounds, the prodigy-LIG server predicted the *K*_D_ for each complex as C1-RBD (−6.73), C2-RBD (−524), C3-RBD (−4.87) and C4-RBD (−6.11), respectively. This demonstrates that these drugs have the ability to suppress the RBD and halt the interaction with hACE2.

## Conclusions

The identification of novel drugs to control the SARS-CoV-2 pandemic is important to overcome the problem of antibodies escaping features of the newly emerging variants. In this regard, computational methods can hasten the identification of such drugs, which could inhibit the variants of SARS-CoV-2. Hence, using virtual screening and simulation approaches potential drugs against the SARS-CoV-2 omicron (B.1.1.529) variant are identified. Simulation and post-simulation validations such as binding free energy calculation, *in silico* bioactivity, and dissociation constant prediction confirmed the potency of these compounds. In conclusion, this study provides a basis for drug designing against the SARS-CoV-2 variants.

## Funding

Dong-Qing Wei is supported by grants from the Key Research Area Grant 2016YFA0501703 of the Ministry of Science and Technology of China, the National Science Foundation of China (Grant No. 32070662, 61832019, 32030063), the Science and Technology Commission of Shanghai Municipality (Grant No.: 19430750600), as well as SJTU JiRLMDS Joint Research Fund and Joint Research Funds for Medical and Engineering and Scientific Research at Shanghai Jiao Tong University (YG2021ZD02).

## Data availability

All the data is available on RCSB, UniProt and any simulation data would be provided on reasonable demand. The accession numbers to access this data are given in the manuscript.

## Conflicts of interest

There are no conflicts of interest.

## Supplementary Material

RA-012-D2RA00277A-s001
