# Supplementary material for: Blocking key mutated hotspot residues in the RBD of the omicron variant (B.1.1.529) with medicinal compounds to disrupt the RBD-hACE2 complex using molecular screening and simulation approaches
Source: RSC Adv. 2022 Mar 4;12(12):7318–27. doi: 10.1039/d2ra00277a (PMC8982251; doi:10.1039/d2ra00277a)
Supplement: RA-012-D2RA00277A-s001 [file RA-012-D2RA00277A-s001.pdf]

Electronic Supplementary Material (ESI) for RSC Advances.
This journal is © The Royal Society of Chemistry 2022

Supplementary Materials

Blocking the Key Mutated hotspot residues in the RBD of Omicron variant (B.1.1.529) with medicinal compounds to disrupt the RBD-hACE2 complex using Molecular screening and Simulation approach

Abbas Khan[1*], AsfandYar Waheed Randhawa[2], Ali Raza Balouch[2], Naila Mukhtar[3], Abrar Mohammad Sayaf[4], Muhammad Suleman[5], Taimoor Khan[1], Shahid Ali[5], Syed, Shujait Ali[5], Yanjing Wang[6*], Anwar Mohammad[7], Dong-Qing Wei[1,8,9*]

[1]Department of Bioinformatics and Biological Statistics, School of Life Sciences and Biotechnology, Shanghai Jiao Tong University, Shanghai, 200240, P.R. China.
[2]Central Park Medical College, Lahore.
[3]Department of Botany, University of Okara, Punjab, Pakistan.
[4]Deparment of Chemistry, University of Swat, Kanju, Khyber Pakhtunkhwa, Pakistan.
[5]Centre for Biotechnology and Microbiology, University of Swat, Kanju, Khyber Pakhtunkhwa, Pakistan.
[6]Engineering Research Center of Cell and Therapeutics Antibody, School of Pharmacy, Shanghai Jiao Tong University, Shanghai, 200240, P.R. China.
[7]Department of Biochemistry and Molecular Biology, Dasman Diabetes Insitutue, Dasman, Kuwait.
[8]State Key Laboratory of Microbial Metabolism, Shanghai-Islamabad-Belgrade Joint Innovation Center on Antibacterial Resistances, Joint Laboratory of International Laboratory of Metabolic and Developmental Sciences, Ministry of Education and School of Life Sciences and Biotechnology, Shanghai Jiao Tong University, Shanghai 200030, P.R. China.
[9]Peng Cheng Laboratory, Vanke Cloud City Phase I Building 8, Xili Street, Nashan District, Shenzhen, Guangdong, 518055, P.R China.

Corresponding Authors

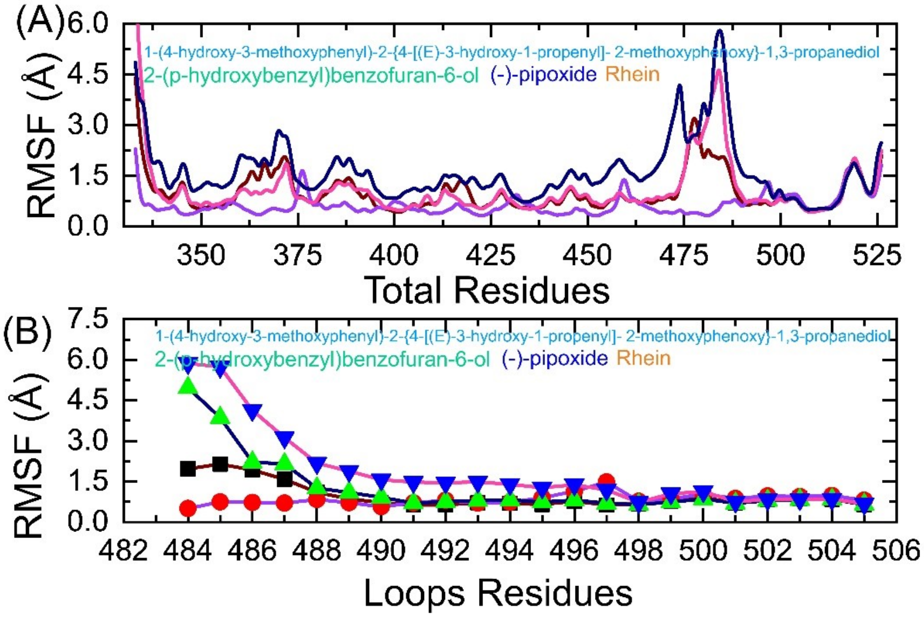

Figure S1: RMSF analysis of the top complexes replica 2. **(A)** Whole structure RMSF, **(B)** loop residues RMSF.

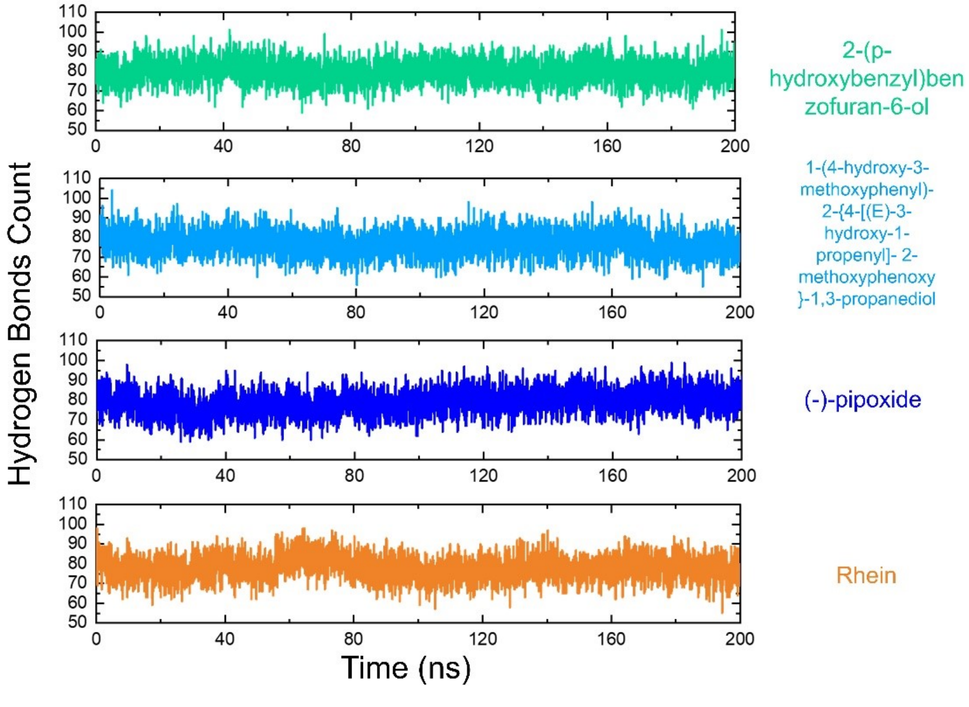

Figure S2: Hydrogen bonding analysis of the top complexes replica 2.
